# Association between Triglyceride to HDL-C Ratio (TG/HDL-C) and Insulin Resistance in Chinese Patients with Newly Diagnosed Type 2 Diabetes Mellitus

**DOI:** 10.1371/journal.pone.0154345

**Published:** 2016-04-26

**Authors:** Xingxing Ren, Zeng.ai Chen, Shuang Zheng, Tingting Han, Yangxue Li, Wei Liu, Yaomin Hu

**Affiliations:** 1 Department of Endocrinology, Renji Hospital, School of Medicine, Shanghai Jiaotong University, Shanghai, China; 2 Department of Radiology, Renji Hospital, School of Medicine, Shanghai Jiaotong University, Shanghai, China; Pennington Biomedical Research Center, UNITED STATES

## Abstract

**Objectives:**

To explore the association between the triglyceride to HDL-C ratio (TG/HDL-C) and insulin resistance in Chinese patients with newly diagnosed type 2 diabetes mellitus.

**Methods:**

Patients with newly diagnosed type 2 diabetes mellitus (272 men and 288 women) were enrolled and divided into three groups according to TG/HDL-C tertiles. Insulin resistance was defined by homeostatic model assessment of insulin resistance (HOMA-IR). Demographic information and clinical characteristics were obtained. Spearman’s correlation was used to estimate the association between TG/HDL-C and other variables. Multiple logistic regression analyses were adopted to obtain probabilities of insulin resistance. A receiver operating characteristic analysis was conducted to evaluate the ability of TG/HDL-C to discriminate insulin resistance.

**Results:**

TG/HDL-C was associated with insulin resistance in Chinese patients with newly diagnosed T2DM (Spearman’s correlation coefficient = 0.21, P < 0.01). Patients in the higher tertiles of TG/HDL-C had significantly higher HOMA-IR values than patients in the lower tertiles [T1: 2.68(1.74–3.70); T2: 2.96(2.29–4.56); T3: 3.09(2.30–4.99)]. Multiple logistic regression analysis showed that TG/HDL-C was significantly associated with HOMA-IR, and patients in the higher TG/HDL-C tertile had a higher OR than those in the lower TG/HDL-C tertile, after adjusting for multiple covariates including indices for central obesity [T1: 1; T2: 4.02(1.86–8.71); T3: 4.30(1.99–9.29)]. Following stratification of waist circumference into quartiles, the effect of TG/HDL-C on insulin resistance remained significant irrespective of waist circumference.

**Conclusions:**

TG/HDL-C was associated with insulin resistance independent of waist circumference. Whether it could be a surrogate marker for insulin resistance in Chinese patients with newly diagnosed type 2 diabetes mellitus still needs to be confirmed by more researches.

## Introduction

Type 2 diabetes mellitus (T2DM) is a progressive disease, characterized by insulin resistance and ongoing loss of endogenous insulin secretion [1]. More and more people are affected by T2DM in recent years. It was reported that the overall prevalence of diabetes in the Chinese adult population was estimated to be 11.6% and the prediabetes was 50.1% in 2013 [2]. T2DM is the most common form of diabetes for Chinese populations, which accounts for more than 90% of all diagnosed diabetes mellitus cases [3]. As insulin resistance is important in the pathogenesis of T2DM, it is helpful to identify early insulin resistance.

The gold standard method for insulin resistance is glucose clamp technique firstly requested by DeFronzo [4]. However, it is complex, time-consuming and invasive, making it not feasible for routine clinical applications. Therefore, large numbers of surrogate indicators have been investigated for insulin resistance. Previous studies have demonstrated that insulin resistance identified by homeostasis model assessment (HOMA-IR) is strongly associated with glucose clamp-assessed insulin resistance [5–7]. This model utilizes a set of empirically derived equations based on the homeostatic concentration of fasting serum insulin (FINS) and fasting plasma glucose (FPG), which reflect the varying degrees of pancreatic β-cell function and insulin resistance [8].

Although HOMA-IR scores are useful indicators of insulin resistance for research purposes, they are expensive and are not readily available to clinicians. Thus, early identification of insulin resistance, preferably by using simple and inexpensive diagnostic tools, is essential for preventing and detecting T2DM. Triglyceride (TG) to high-density lipoprotein cholesterol (HDL-C) ratio has been proposed as a simple marker of insulin resistance [9–14]. The potential utility of TG/HDL-C to detect insulin resistance was firstly reported by McLaughlin in a Caucasian population [10]. Similar results were found in different racial groups such as Korean [15], non-Hispanic Black and Mexican American [16]. However, studies showed that TG/HDL-C might not be a marker of insulin resistance for African populations [17–20]. It is possible that given the racial variations in both TG and HDL-C levels, the association between TG/HDL-C and insulin resistance is ethnicity-dependent. There are limited evidences supporting that the ratio of TG/HDL-C is a surrogate marker of insulin resistance in Chinese individuals [14, 21–23]. What’s more, few studies have been conducted in newly diagnosed T2DM patients. Thus, this study focused on the plasma lipid profiles and explored the association between TG/HDL-C and insulin resistance in Chinese patients with newly diagnosed T2DM.

## Methods

### Study populations

Participants were recruited when they were diagnosed with T2DM for the first time at Renji Hospital located in Shanghai, China between January 2008 and December 2009. The diagnostic criteria for diabetes mellitus was the one that promulgated by World Health Organization (WHO) in 1999 [24]. Newly diagnosed T2DM patients who had never taken medicine for prediabetes and dyslipidemia diseases were enrolled in the study. Exclusion criteria included (1) the presence of diabetes or other chronic diseases such as heart and kindey diseases, (2) the use of medications that may affect blood pressure, glucose, or lipid metabolism, (3) thyroid and other endocrine diseases, (4) serious infections and trauma, (5) stress (6) and the informed consent form not being signed. The correlation coefficient between the TG/HDL -C ratio and HOMA-IR was 0.33–0.41 in a Chinese population [23]. The study’s target sample size was at least 92 or 58 by using Power Analysis and Sample Size 11 (PASS 11, a professional software for sample size calculation in clinical trials). 560 patients (272 men and 288 women) with newly diagnosed T2DM were involved in this study. Patients were divided into three groups according to tertiles of TG/HDL-C: T1: 0.12–0.95 (n = 186); T2: 0.96–1.70 (n = 186); T3: 1.71–10.14 (n = 188). All work was carried out in compliance with the declaration of Helsinki. The study protocol was approved by the Ethical Committee of Renji Hospital, School of Medicine, Shanghai Jiaotong University, Shanghai, China (Number: Renjikls N026). All participants provided written informed consent prior to enrollment.

### Data collection

Anthropometric measurements including height, weight, waist circumference (WC) and hip circumference (HC) were measured by the WHO recommended protocols [25]. Body mass index (BMI) was calculated using the ratio of weight/height ^2^ (kg/m^2^). Waist to hip ratio (WHR) and waist to height ratio (WHtR) were calculated as WC divided by HC and height, respectively. Blood pressure (BP) was measured three times and the average of the three consecutively measurement was calculated [26]. All the patients were asked to be on a low-fat diet and avoid strenuous exercise before being taken blood samples. Following a 12-hour overnight fast, blood samples were collected from the antecubital vein of patients. Various biochemical markers including alanine transaminase (ALT), glycated hemoglobin A1c (HbA1c), total cholesterol (TC), TG, HDL-C, low-density lipoprotein cholesterol (LDL-C) were obtained. Standard oral glucose tolerance tests (75 g glucose load) and insulin releasing tests were conducted. Levels of plasma glucose and serum insulin were detected at 0, 30, 60, 120, 180 minutes of the tests. G_0_, G_30_, G_60_, G_120_, G_180_ symbolized the plasma glucose levels of the corresponding time. Similarly, INS_0_, INS_30_, INS_60_, INS_120_, INS_180_ denoted the serum insulin levels. G_0_ stood for FPG, and INS_0_ was on behalf of FINS.

Insulin resistance was determined using HOMA-IR. The following equation was used for HOMA-IR index: INS_0_ (IU/L) ×G_0_ (mmol/L)/22.5 [8]. Based on an epidemiology survey in China, insulin resistance was defined as HOMA-IR > 2.69 [27]. Homeostatic model assessment of β-cell function (HOMA-β) was from this equation: 20 ×INS_0_ (IU/L)/(G_0_ (mmol/L)-3.5) [6]. Insulin sensitivity was evaluated by insulin sensitivity index (ISI) composite: 10,000/([G_0_ × INS_0_] × [mean G_0-120_ × mean INS_0-120_] (mean G_0-120_ refers to the average plasma glucose level for the first 120 minutes calculated by the average value of G_0_, G_30_, G_60_ and G_120_) [28]. Early insulin secretion was calculated based on the formula for insulinogenic index (IGI, ΔINS_30_/ΔG_30_): (INS_30_-INS_0_) (IU/L)/(G_30_-G_0_) (mmol/L) [29].

### Statistical analysis

Descriptive statistics for continuous variables were presented as mean ± standard deviation if variables were normally distributed, or as median and interquartile range (25%-75%) for skewed variables. Independent-samples t test and one-way analysis of variance test were adopted for normally distributed data. If the data showed a skewed distribution, Mann-Whitney U test and Kruskal-Wallis H test were taken for differences. Pearson’s correlations were looked into to measure the associations between TG/HDL-C and other variables when they were normally distributed, if not, Spearman’s correlations were adopted. To figure out the clinical utility of TG/HDL-C in predicting insulin resistance, the receiver operating characteristic (ROC) curve was plotted and the area under the curve was calculated.

The primary focus of the analysis was to investigate the association between insulin resistance and TG/HDL-C values as risk factors for T2DM. For the odds ratios (ORs) and 95% confidence intervals (95% CIs) of TG/HDL-C for insulin resistance, the stepwise logistic regression analyses were performed to examine the relationship between insulin reisistance as the dependent variable and TG/HDL-C, by controlling for confounding variables including sex, age, SBP, BMI, ALT, and HbA1c. The previous study indicated central obesity was a risk factor for insulin resistance [30]. Hence, we added indicators which stood for central obesity including WC, WHR and WHtR in the models. To further evaluate the interactive effect of the TG/HDL-C ratio by WC on insulin resistance, WC was stratified into four groups based on quartiles. The quartiles of WC were categorised as follows: Q1: 63.0–83.0cm; Q2: 83.1–90.0cm; Q3: 90.1–98.0cm; Q4:98.1–121.0cm. Multiple logistic regression analysis was employed to analyze the interactions among WC, TG/HDL-C and insulin resistance. Each combination was compared to the composite of the first tertile of TG/HDL-C and the first quartile of WC.

All statistical analyses were performed using the SPSS statistical package (version 20.0, SPSS Inc, USA). All statistical tests were two-sided, and a P-value < 0.05 was considered statistically significant.

## Results

### Comparisons of the general characteristics among patients of newly diagnosed T2DM with different tertiles of TG/HDL-C

Clinical data and baseline characteristics of patients with different tertiles of TG/HDL-C are presented in Table 1. Patients in the higher tertiles of TG/HDL-C had higher DBP, BMI, WC, WHR, WHtR, TG, TC, Log(TG/HDL-C), HbA1c, FPG, FINS, and HOMA-IR than patients in the lower tertiles of TG/HDL-C, and the significant differences were mainly between T1 and T2, T1 and T3. Yet, HDL-C and ISI concentration decreased significantly. Mean values of SBP, HOMA-β, and IGI were not significantly different among different TG/HDL-C tertiles.

**Table 1:** Clinical and metabolic characteristics of patients according to TG/HDL-C tertiles.

Variables	T1 (0.12–0.95) n = 186		T2 (0.96–1.70) n = 186		T3 (1.71–10.14) n = 188	
Age (year)	56(50.50–62.50)		56(47.50–63)		54(45.25–60)*#	
SBP (mmHg)	130(120–140)		130(120–140)		130(120–142)	
DBP (mmHg)	80(72–89)		80(78.50–89.50)*		84(80–90)*	
BMI (kg/m^2^)	24.13(22.24–26.42)		25.29(23.52–27.20)*		25.65(23.56–28.06)*	
WC (cm)	M87(79–93)	F84(76–88)	M91.5(88–97)*	F86(79–92)*	M92(88–96)*	F86(81–94)*
WHR	0.88(0.85–0.93)		0.91(0.87–0.96)*		0.93(0.89–0.96)*	
WHtR	0.52(0.48–0.55)		0.54(0.51–0.57)*		0.54(0.51–0.58)*	
ALT (U/L)	21(15–33.25)		30.50(20–42.50)*		29(19–53)*	
TG (mmol/L)	0.97(0.81–1.20)		1.58(1.36–1.85)*		2.67(2.17–3.35)*#	
TC (mmol/L)	4.88(4.20–5.54)		5.13(4.49–5.80)*		5.13(4.46–5.87)*	
LDL-C (mmol/L)	2.97(2.46–3.46)		3.33(2.82–3.81)*		3.23(2.67–3.72)*	
HDL-C (mmol/L)	1.51(1.34–1.81)		1.28(1.13–1.42)*		1.07(0.96–1.24)*#	
Log(TG/HDL-C)	-0.17(-0.28–0.86)		0.10(0.03–0.15)*		0.37(0.28–0.50)*#	
HbA1c (%)	6.55(5.98–7.50)		6.90(6.28–7.73)*		6.90(6.20–8.58)*	
FPG (mmol/L)	6.70(6.10–7.53)		7.00(6.38–7.90)		7.10(6.23–8.52)*	
2hPG (mmol/L)	13.30(11.48–15.55)		13.70(11.60–16.70)		13.90(11.63–17.20)	
FINS (μIU/ml)	8.63(6.03–11.48)		9.68(7.39–13.59)*		10.14(7.17–14.56)*	
2hPINS (μIU/ml)	42.61(26.17–75.62)		51.02(31.51–78.26)		50.95(25.90–84.90)	
HOMA-IR	2.68(1.74–3.70)		2.96(2.29–4.56)*		3.09(2.30–4.99)*	
HOMA-β	51.20(34–76.41)		58.17(35.18–87.11)		55.45(34.28–84.10)	
ISI	0.53(0.29–1.05)		0.39(0.20–0.66)*		0.35(0.19–0.69)*	
IGI	2.71(1.16–5.40)		3.13(1.07–5.68)		3.17(1.02–6.41)	

Abbreviations: SBP:systolic blood pressure; DBP:diastolic blood pressure; BMI:body mass index; WC:waist circumference; M:male; F:female; WHR:waist to hip ratio; WHtR: waist to height ratio; ALT:alanine transaminase; TG:triglyceride; TC:total cholesterol; LDL-C: low-density lipoprotein cholesterol; HDL-C:high-density lipoprotein cholesterol; HbA1c: glycated hemoglobin A1c; FPG: fasting plasma glucose; 2hPG:2-hour postprandial plasma glucose; FINS:fasting serum insulin; 2hPINS:2-hour postprandial serum insulin; HOMA-IR:homeostatic model assessment of insulin resistance; HOMA-β:homeostatic model assessment of β-cell function; ISI:insulin sensitivity index; IGI: insulinogenic index.

* means comared to T1, P < 0.05.

^#^ means compared to T2, P < 0.05.

### Gender-adjusted Spearman’s correlations between other variables and TG/HDL-C

Gender-adjusted Spearman’s correlations between other variables and TG/HDL-C are shown in Table 2. TG and HDL-C were not analyzed as they were included in the formula of TG/HDL-C value. We just listed the data which were statistically significant. As is shown in Table 2, DBP, BMI, WC, WHR, HbA1c, Log(TG/HDL-C), FPG, FINS, HOMA-IR were positively associated with TG/HDL-C (Spearman’s correlation coefficient = 0.15, 0.21, 0.25, 0.26, 0.18, 1, 0.16, 0.16, 0.21, P < 0.01). Negative association was seen between ISI and TG/HDL-C (Spearman’s correlation coefficient = -0.18, P < 0.01). Some variables such as SBP, LDL-C, ALT and 2hPINS were positively associated with TG/HDL-C. Unfortunately, these associations were not statistically significant (data were not shown).

**Table 2:** Gender-adjusted Spearman’s correlations between TG/HDL-C and other variables.

Variables	TG/HDL-C	p
DBP	0.15	0.001
BMI	0.21	< 0.001
WC	0.25	< 0.001
WHR	0.26	< 0.001
HbA1c	0.18	< 0.001
Log(TG/HDL-C)	1	
FPG	0.16	< 0.001
FINS	0.16	< 0.001
HOMAIR	0.21	< 0.001
ISI	-0.18	< 0.001

Abbreviations: DBP:diastolic blood pressure; BMI:body mass index; WC:waist circumference; WHR:waist to hip ratio; HbA1c:glycated hemoglobin A1c; FPG: fasting plasma glucose; FINS:fasting serum insulin; HOMA-IR:homeostatic model assessment of insulin resistance; ISI:insulin sensitivity index.

### Stepwise logistic regression analyses for insulin resistance

Stepwise logistic regression analyses designed to examine the relationship between the tertiles of TG/HDL-C and insulin resistance are listed in Table 3. The variables including sex, age, SBP, BMI, ALT, and HbA1c were adjusted for in the logistic regression analysis to calculate ORs in Model 1. In order to control central obesity, three more variables including WC, WHR and WHtR were added in Model 4 to investigate the effect of the TG/HDL-C ratio on insulin resistance. Patients in the higher TG/HDL-C tertile had a higher OR compared to those in the lower quartile [T1: 1; T2: 4.02(1.86–8.71); T3: 4.30(1.99–9.29)]. All these ORs were statistically significant. Moreover, our data demonstrated that WC and WHR might be important interference factors because they were still in the regression equation at last.

**Table 3:** Multivariable-adjusted odds ratios for insulin resistance according to each tertile of TG/HDL-C.

	TG/HDL-C	T1(0.12–0.95)	T2(0.96–1.70)	T3(1.71–10.14)
**Model 1**	OR(95% CI)	1	3.00(1.50–6.02)	3.11(1.56–6.22)
**Model 2**	OR(95% CI)	1	3.30(1.58–6.89)	3.53(1.68–7.41)
**Model 3**	OR(95% CI)	1	4.02(1.86–8.71)	4.30(1.99–9.29)
**Model 4**	OR(95% CI)	1	4.02(1.86–8.71)	4.30(1.99–9.29)

Model 1: Adjusted for sex, age, SBP, BMI, ALT, HbA1c.

Model 2: Adjusted for WC, in addition to the factors in Model 1.

Model 3: Adjusted for WHR, in addition to the factors in Model 2.

Model 4: Adjusted for WHtR, in addition to the factors in Model 3.

Abbreviations: TG/HDL-C:triglyceride to high-density lipoprotein cholesterol ratio; T:tertile. SBP:systolic blood pressure; BMI:body mass index; ALT:alanine transaminase; HbA1c:glycated hemoglobin A1c; WC:waist circumference; WHR:waist to hip ratio; WHtR: waist to height ratio.

### ROC curves of TG/HDL-C, TG and HDL-C for insulin resistance

**Fig 1:**
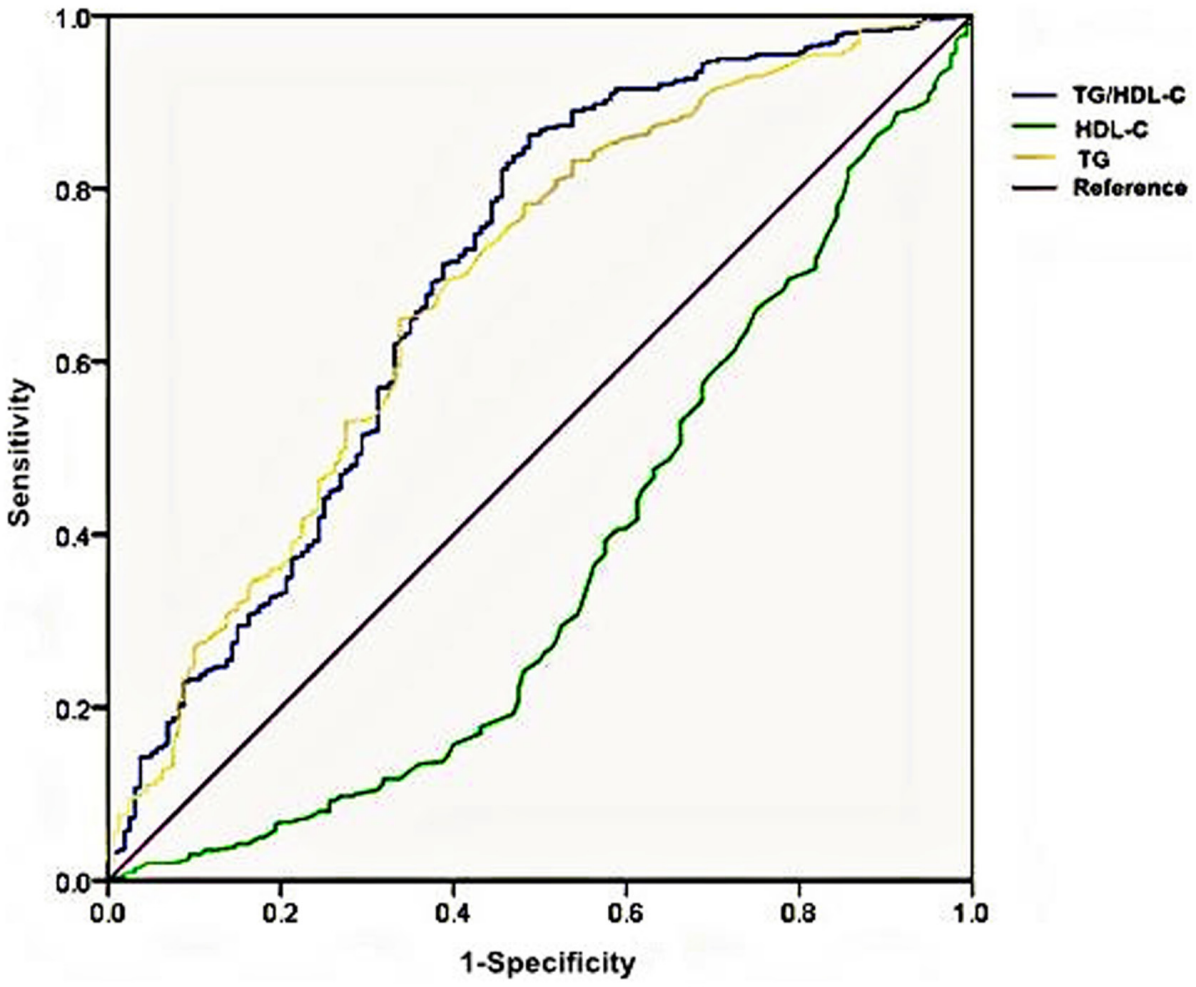
Receiver operating characteristic (ROC) curves of TG/HDL-C, TG, and HDL-C for insulin resistance. The area under the ROC curve ± standard error (95% CI):TG/HDL-C: 0.70 ± 0.03(0.65–0.75); TG: 0.69 ± 0.03(0.64–0.74); HDL-C: 0.37 ± 0.03(0.31–0.42). Abbreviations: TG:triglyceride; HDL-C:high-density lipoprotein cholesterol; TG/HDL-C:triglyceride to high-density lipoprotein cholesterol ratio.

In order to compare the efficiency for insulin resistance among TG/HDL-C, TG and HDL-C, the ROC curves were plotted (see in Fig 1). For the TG/HDL-C ratio, an incremental increase in true-positive rates (sensitivity) was associated with relatively smaller increases in false positive rates (1-specificity) as compared with the curves of TG or HDL-C. In addition, the TG/HDL-C ratio presented the greatest value of the area under the ROC curve with statistical significance [TG/HDL-C:0.70 ± 0.03(0.65–0.75); TG: 0.69 ± 0.03(0.64–0.74); HDL-C: 0.37 ± 0.03(0.31–0.42)].

### Combinations of TG/HDL-C tertiles and WC quartiles to examine the interactive effect on insulin resistance

**Fig 2:**
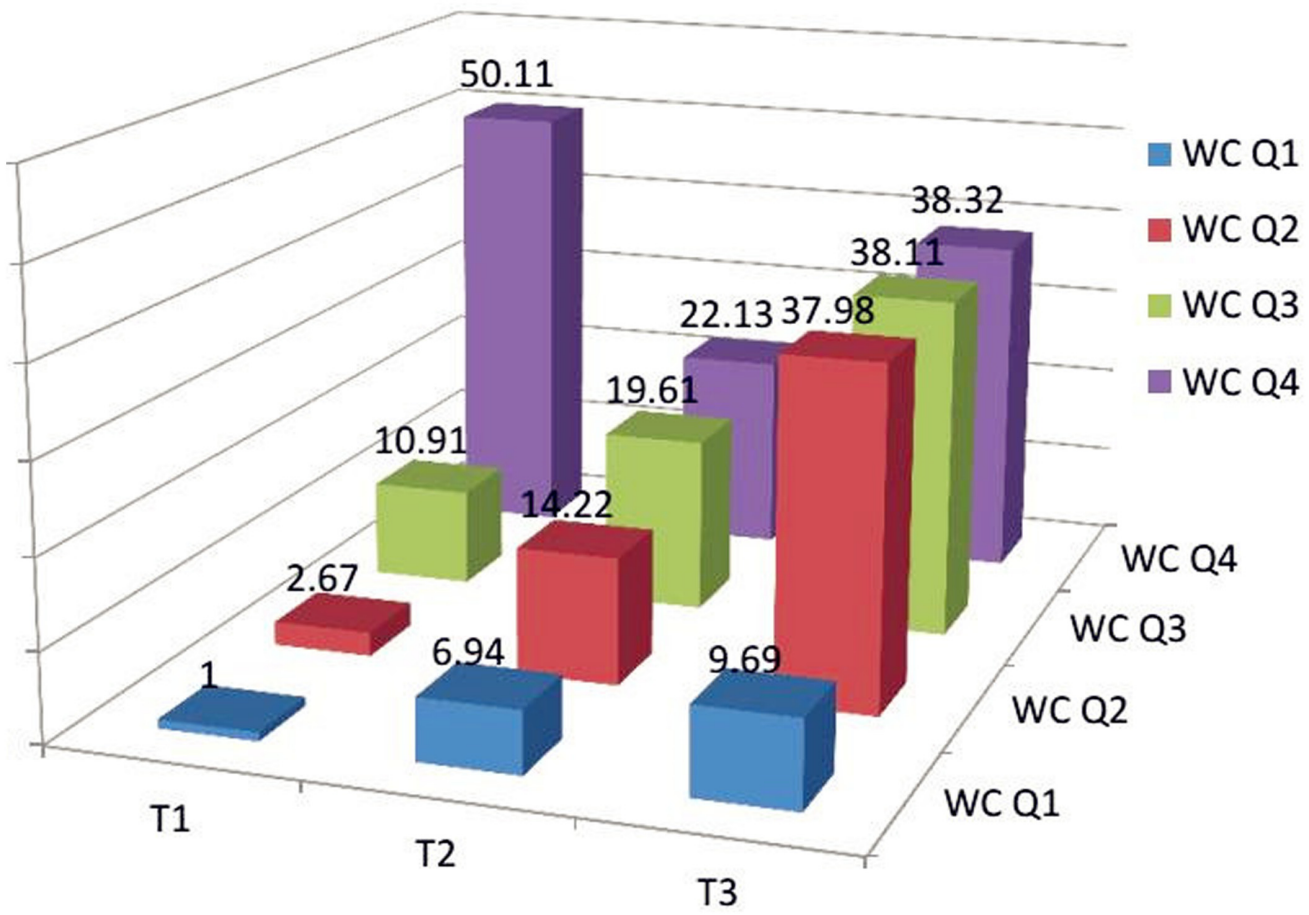
The relationship with insulin resistance of each combination of TG/HDL-C tertiles and WC quartiles. Each block of OR represents the OR as against the OR which the patients belonging to the first tertile of TG/HDL-C and the first quartile of WC. Abbreviations: WC:waist circumference; T:tertile; Q:quartile.

We combined TG/HDL-C tertiles and WC quartiles which were cross-tabulated to examine the interactive effect on insulin resistance (see in Fig 2). Each composite had a higher OR than the combination of the first tertile of TG/HDL-C and the first quartile of WC. Provided that the OR of the first combination was one, more than half of the ORs were larger than ten. The figure showed that TG/HDL-C had an increasing association with insulin resistance in any WC quartile except WC Q4, and all the data were statistically significant. In addition, given the same TG/HDL-C tertile such as T3, the ORs of WC for insulin resistance presented an increasing pattern of association.

## Discussion

In this study, we found that TG/HDL-C was associated with insulin resistance in Chinese patients with newly diagnosed T2DM. The association still existed regardless of WC, WHR and WHtR after adjusting for sex, age, SBP, BMI, ALT, and HbA1c. Furthermore, HOMA-IR increased with the rising TG/HDL-C levels. In Giannini’s study, modest correlations were found between the TG/HDL-C ratio and insulin sensitivity measured by the hyperinsulinemic-euglycemic clamp, what’s more, TG/HDL-C was a more meaningful indicator than HOMA-IR [31].

High TG and low HDL-C levels are known to be associated with insulin resistance and T2DM [32] and cardiovascular diseases (CVDs) [33]. In addition, they have been explored to be used for insulin resistance, but the discriminatory power of single TG or HDL-C was poor, and TG/HDL-C was a stronger risk factor [34]. This is consistent with our study. As is shown in Fig 1, TG/HDL-C had the largest area of ROC compared with TG and HDL-C, reaching diagnostic significance. This is similar to the results of Korean studies [15, 35]. Zhang et al. [23] got an 0.73 area of TG/HDL-C ROC in overweight/obese women, which was a little larger than ours. Although both of us studied on Chinese populations, distinct physical or health condition was the main difference. Giannini et al. [31] used the hyperinsulinemic-euglycemic clamp data as the gold standard of insulin sensitivity, the evaluation of TG/HDL-C was significant for the whole cohort regardless of ethnicity and sex (0.752 ± 0.042, P < 0.001).

Central obesity is one of the strongest indicators of insulin resistance. WC, WHR and WHtR are the useful measurements for central obesity, and WC is the most sensitive index [36]. It has been reported that individuals with higher WC have more visceral fat and are more susceptible to chronic diseases caused by insulin resistance than those with lower WC and the same BMI [37]. The effects of WC and other indices on insulin resistance should be considered when examining the relationship between the TG/HDL-C ratio and insulin resistance. Therefore, we added WHR, WHtR, and WC to the models one by one. Table 3 is the tabulation of odds ratio (OR) across all tertiles of TG/HDL-C ratio in relation to insulin resistance. Patients in the higher TG/HDL-C tertile had a higher OR compared to those in the lower tertile. In order to eliminate the independent effect of WC on insulin resistance, stratification of WC was conducted. Fig 2 shows that TG/HDL-C was still allowed to maintain statistically significant relationship with insulin resistance. All the other eleven combinations had a higher OR than the combination of first tertile of TG/HDL-C and the first quartile of WC. Where, at a given WC quartile, patients in the higher tertile of the TG/HDL-C ratio were more likely to be insulin resistant in relation to those in the lower quartiles except the last quartile of WC. Korean studies [15, 35] adopted the stratification of WC to dispel its effect on insulin resistance as well. One also had exceptional conditions which were analogous to ours [15]. The other indicated that participants in the higher quartile of TG/HDL-C had a higher OR than those in any lower quartile independent of WC [35]. That our sample is not large enough may account for this exception. As is shown in Fig 2, patients in the third tertile of TG/HDL-C were found to be so strongly associated with the prevalence of insulin resistance, that these ORs were about thrice the values of these in T1. Moreover, in some given WC quartile, the ratio reached more than nine.

In our previous clinical studies, we found increased plasma triglyceride levels were associated with T2DM, which contributed to cardiovascular diseases [38, 39]. Lipoprotein lipase gene mutations may play an important role in dyslipidemia in T2DM patients [38]. Elevated insulin resistance and TGs are both independently and inversely relevant to reduced HDL-C [40]. The TG/HDL-C ratio may serve as a more appropriate value of insulin resistance when predicting CVD-related morbidity and mortality [41–43]. Dobiasova et al. [44] revealed that Log(TG/HDL-C) had a strong association with the diameter of LDL-C particle, and named it atherogenic index of plasma (AIP), suggesting it as an indirect index for the diameter of LDL-C particle. As the TG/HDL-C ratio increases, LDL-C particles become smaller and denser, which contribute to the development of atherosclerosis and CVDs [45–48]. Barter et al. [49] demonstrated that higher levels of TG/HDL-C ratio were correlated with greater potential for developing CVDs through insulin resistance and more atherogenic LDL-C particles, even when LDL-C levels were in the normal range. A American study [50] revealed that individuals with higher TG/HDL-C had significantly higher BP, a more adverse lipid profile and was more insulin resistant as assessed by HOMA-IR. They drew the conclusions that TG/HDL-C provided a simple approach to identify individuals at a accentuated cardio-metabolic risk within a population of perceived increased risk of T2DM. In our study, we found that with the increasing tertile, DBP ascended as well. In addition, positive association was found between DBP and TG/HDL-C. Patients in the higher tertile had a more unfavorable lipid profile, especially for TG and HDL-C, which were consistent with Armato’s study [50]. Given the many syndromes associated with insulin resistance, including T2DM and coronary heart diseases, an elevated TG/HDL-C ratio supports more aggressive efforts to enhance insulin sensitivity.

We found that as TG/HDL-C increased, FPG and FINS increased as well. This bore an analogy to the result from Hirschler et al. [11]. 2hPG was increasing, but the differences were not statistically important. 2hPINS presented an increasing curve but decreased in the third tertile. As TG/HDL-C increased, HOMA-IR increased and ISI decreased. A American study also found that across the tertile groups, ISI significantly decreased and HOMA-IR significantly increased [31]. Patients in T2 and T3 groups reached insulin resistance according to the average level of HOMA-IR. As is shown in Table 2, FPG and FINS were positively associated with TG/HDL-C ratio. In our study, we found HOMA-β among different TG/HDL-C tertiles presented a wave, and IGI was on the rise. However, differences did not have statistical significance. These were similar to our another study [51]. Simental-Mendia et al. [52] promulgated that elevated TG levels were associated with HOMA-IR and HOMA-β indices in healthy children and adolescents with normal weight in Mexico, HOMA-β decreased as the TG/HDL-C ratio increased. Hermans et al. [53] reported that log(TG)/HDL-C was related to both residual cardio-metabolic risk and β-cell function loss in males with type 2 diabetes mellitus. These results were different from those of ours. Some causes led to these differences. Their studying subjects were from different races compared with ours, in addition, their patients were with normal weight. Besides, our patients were newly diagnosed with T2DM, the function of pancreatic β-cell was still in the compensatory phase.

There are certain limitations that should be noted here. Firstly, the sample of the present study was not large enough and the patients were from the areas surrounding Shanghai city, thus it can not symbolize the general situation. Secondly, we did not supply the comparison of TG/HDL-C with the gold standard. Last but not least, we didn’t offer the cut-off of TG/HDL-C for insulin resistance.

## Conclusions

In conclusion, this study demonstrated that TG/HDL-C was associated with insulin resistance in Chinese patients with newly diagnosed T2DM. After adjusting for confounding variables, the association still existed regardless of BMI, WC, WHR and WHtR. HOMA-IR went up with the increase of TG/HDL-C. In addition, the TG/HDL-C ratio may serve as a more appropriate value of insulin resistance when predicting CVD-related morbidity and mortality. Whether this criterion (TG/HDL-C) could be an easy and inexpensive marker of insulin resistance for newly diagnosed T2DM still needs to be confirmed by more researches.

## Supporting Information

S1 File This file contains the original data. (XLS)
